# Abstracts from the 4th Global Chinese Symposium and the 8th Symposium for cross-straits, Hong Kong and Macao on free radical biology and medicine

**DOI:** 10.1186/s13020-018-0212-y

**Published:** 2018-11-26

## A1 Peroxynitrite-mediated mitophagy could be a crucial therapeutic target for reducing cerebral ischemia–reperfusion injury

### Jiangang Shen, Jinghan Feng, Hansen Chen

#### School of Chinese Medicine, Li Ka Shing Faculty of Medicine, The University of Hong Kong, 10 Sassoon Road, Hong Kong, SAR, China

##### **Correspondence:** Jiangang Shen - shenjg@hku.hk

*Journal of Chinese Medicine* 2018, **13(Supp 2):**A1

Basic autophagy/mitophagy is essential for cell survival whereas excessive autophagy/mitophagy is detrimental during cerebral ischemia–reperfusion (I/R) injury. Peroxynitrite (ONOO^−^), a representative of reactive nitrogen species, is a critical neurotoxic factor in mediating cerebral I/R injury, but its roles in autophagy/mitophagy remain unclear. Herein, we hypothesized that ONOO^−^ could induce PINK1/Parkin-mediated mitophagy activation via triggering dynamin-related protein 1 (Drp1) recruitment to damaged mitochondria, contributing to cerebral I/R injury. The major discoveries revealed that: (1) PINK1/Parkin-mediated mitophagy activation was predominant among general autophagy, leading to rat brain injury at the reperfusion phase after cerebral ischemia; (2) increased nitrotyrosine was found in the plasma of ischemic stroke patients and ischemia–reperfused rat brains, indicating the generation of ONOO^−^ in ischemic stroke; (3) ONOO^−^ was dramatically increased in accompanied with mitochondrial recruitment of Drp1, PINK1/Parkin-mediated mitophagy activation, and progressive infarct size in rat ischemic brains at the reperfusion phase; (4) FeTMPyP, a peroxynitrite decomposition catalyst, remarkably reversed mitochondrial recruitment of Drp1, mitophagy activation and brain injury; (5) ONOO^−^ induced tyrosine nitration of Drp1 peptide and mitochondrial recruitment of Drp1 for mitophagy activation. Those results suggest that ONOO^−^-induced mitophagy activation aggravates cerebral I/R injury via recruiting Drp1 to damaged mitochondria.

Furthermore, we investigated ONOO^−^-induced mitophagy as a therapeutic target for attenuating cerebral I/R injury by using a natural antioxidant naringin as an example. Naringin possessed strong ONOO^−^ scavenging capability and inhibited the production of superoxide and nitric oxide in SH-SY5Y cells under 10 h oxygen-glucose-deprivation plus 14 h of reoxygenation or ONOO^−^ donor 3-morpholinosydnonimine conditions. Naringin also inhibited NADPH oxidases and iNOS in rat brains with 2 h ischemia plus 22 h reperfusion. Naringin was able to cross the blood–brain barrier, decreased neurological deficit score and infarct size, and attenuated apoptotic cell death. Naringin reduced 3-nitrotyrosine formation, decreased the ratio of LC3-II to LC3-I in mitochondrial fraction, and inhibited the translocation of Parkin to the mitochondria. Taken together, peroxynitrite-mediated mitophagy activation could be a potential therapeutic target for cerebral I/R injury.

## A2 Dual roles of antioxidant enzymes: mechanism and implication

### Xin Gen Lei^1^, Jian-Hong Zhu^2^

#### ^1^Department of Animal Science, Cornell University, Ithaca, New York, USA; ^2^Department of Preventive Medicine, Wenzhou Medical University, Wenzhou, Zhejiang, China

##### **Correspondence:** Xin Gen Lei - xl20@cornell.edu

*Journal of Chinese Medicine* 2018, **13(Supp 2):**A2

**Background:** Reactive oxygen species (ROS) and reactive nitrogen species (RNS) are generated from aerobic metabolism, as a result of accidental electron leakage as well as regulated enzymatic processes. Because ROS/RNS can induce oxidative injury and act in redox signaling, enzymes metabolizing them will inherently promote either health or disease, depending on the physiological context. It is thus misleading to consider conventionally called antioxidant enzymes to be largely, if not exclusively, health protective. As such a notion that antioxidant enzymes are beneficial is common, we herein attempted to rationalize why this simplistic view should be avoided [1].

**Materials and methods:** Physiological phenotypes triggered in mouse models of overexpression or knockout of major antioxidant enzymes are reviewed and summarized. Mechanisms by which these phenotypes are mediated are elaborated with regard to chemical, biological, and metabolic interactions of the antioxidant enzymes with their substrates, downstream events, and cellular context.

**Results:** Paradoxical functions in association with metabolism, health, and diseases exist in superoxide dismutases, catalase, glutathione peroxidases, thioredoxin reductases, thioredoxins, glutaredoxins, and peroxiredoxins, along with other selenoproteins and selenoprotein synthesis-related selenocysteine tRNA. Novel treatments of antioxidant enzyme-related human diseases are proposed by deliberate targeting of dual roles of the pertaining enzymes. Paradoxical roles of antioxidant enzymes derive from sophisticated molecular mechanisms of redox biology and metabolic homeostasis.

**Conclusions:** Simply viewing antioxidant enzymes as always being beneficial is not only conceptually misleading but also clinically hazardous if such notions underpin medical treatment protocols based on modulation of redox pathways.

**Reference**

1. Lei XG, Zhu JH, Cheng WH, Bao Y, Ho YS, Reddi AR, Holmgren A, Arner ESJ. Paradoxical roles of antioxidant enzymes: Basic mechanisms and health implications. Physiol Rev. 2016;96:307–64.

## A3 To explore the mechanism of inflammation and degeneration of the central nervous system: role of brain astrocytes

### Hsi-Lung Hsieh^1,2^

#### ^1^Department of Nursing, Division of Basic Medical Sciences, Research Center for Chinese Herbal Medicine, and Graduate Institute of Health Industry Technology, Chang Gung University of Science and Technology, Tao-Yuan, Taiwan; ^2^Department of Neurology, Chang Gung Memorial Hospital, Tao-Yuan, Taiwan

##### **Correspondence:** Hsi-Lung Hsieh - hlhsieh@mail.cgust.edu.tw

*Journal of Chinese Medicine* 2018, **13(Supp 2):**A3

**Background:** Inflammation is a central pathogenic mechanism of various neuropathies including neurodegenerative diseases. In chronic neurodegenerative diseases such as Alzheimer’s disease (AD), the pathology is associated with an abnormal inflammatory response, characterized by the activation of several cell populations in the brain such as neuroglial cells. The relationships between inflammation and the development of these neuropathies involve complex molecular networks and processes. One common feature of various neurodegenerative diseases is activation of large number of astrocytes and microglia that includes the morphological changes and expression of many inflammatory mediators. Increasing studies have indicated that the cell–cell interactions between glial cells and neurons may be important in the regulation of brain inflammation and neurodegeneration. Thus, these results implicate that activated neuroglial cells, astrocytes especially, play a critical role in the progression and pathogenesis of neurodegenerative disorders. Moreover, recent evidence suggests that brain inflammation may impact on local inflammation in the brain diseases leading to over-production of several inflammatory mediators, which may in turn influence functions including apoptosis. Elevated levels of several pro-inflammatory factors including cytokines, peptides, pathogenic structures, per-oxidants in central nervous system (CNS) have been detected in the patients with brain disorders like AD. These pro-inflammatory factors exert as potent stimuli in brain inflammatory responses through up-regulation of diverse inflammatory mediators.

**Results and conclusions:** Here, we explored the mechanisms underlying the intracellular signaling pathways (e.g., protein kinase Cs, reactive oxygen species, or mitogen-activated protein kinases) involved in the expression of inflammatory mediators induced by pro-inflammatory factors in brain astrocytes and its effects on neuronal cells. Understanding of the regulatory mechanisms involved in the relationship of neuroglia and neuronal cells may provide the helpful therapeutic strategy for brain injury, inflammation, and neurodegenerative disorders.

**Acknowledgements:** We thank funding grants from the Ministry of Science and Technology, Taiwan (NSC102-2320-B-255-005-MY3; MOST106-2320-B-255-005; MOST107-2320-B-255-003) and Chang Gung Medical Research Foundation (CMRPF1C0193; CMRPF3D0033; CMRPF1F0131; CMRPF1F0132; CMRPF1H0051).

**References**

1. Hsieh HL, et al. Role of redox signaling in neuroinflammation and neurodegenerative diseases. Biomed Res Int. 2013;2013:484613–30.
2. Hsieh HL, et al. The role of matrix metalloproteinase-9 in pro-inflammatory factors-induced brain inflammation and neurodegenerative diseases. Inflamm Cell Signal. 2014;1:88–96.
3. Hsieh HL, et al. Astrocyte as the modulator in brain inflammation and neurodegenerative disorders. Ann Neurodegener Dis. 2016;1(2):1008.

## A4 Evaluation of the Ganoderma effects in pain sensitivity of aging mice with the homemade tail flick instrument

### Chia-Ying Lin^1^, Ming-Wei Chao^2^, Wei-Nong Li^1^, Chao-Ming Tang^1^, Chia-Yi Tseng^1^

#### ^1^Biomedical Engineering, Chung Yuan Christian University, Zhongli District, Taoyuan City, 320, Taiwan; ^2^Bioscience Technology, Chung Yuan Christian University, Zhongli District, Taoyuan City, 320, Taiwan

##### **Correspondence:** Chia-Yi Tseng - cytseng@cycu.edu.tw

^*^Chia-Ying Lin and Ming-Wei Chao contributed equally to this work

*Journal of Chinese Medicine* 2018, **13(Supp 2):**A4

**Background:** It is become more important on elderly care because the aging of population. It also leads to the increase of developing health-care products, such as Monacolin, fish oil, Lactobacillus and plant sterols etc. *Ganoderma tsugae* have ingredients including polysaccharide, triterpenes, adenosine and small-molecule proteinase. *Ganoderma tsugae* is attributed with therapeutic properties, such as anticancer, regulate blood sugar, antioxidation, antibiosis, antivirus, liver-protecting and protect stomach damage. Normal physiological pain is a type of admonition for human to perceive harmful stimulate and take enough response time to avoid further damage. There are some research show that increasing oxidative stress in elder’s brain is one of the reason of decreasing pain sensitivity. Thus elder have lower physiological pain than youth, it may increase the possibility of suffer injury. So, we suppose that *Ganoderma tsugae* can reduce the oxidative stress in elder’s brain and increase their pain sensitivity.

**Materials and methods:** We gave d-galactose (100 mg/kg B.W./day) by injection to induce mice aging. And *Ganoderma tsugae* (200 μg/kg B.W./day) was gave by oral gavage. We measured the pain sensitivity of galactose-induced aging mice with formalin test, hot water test and self-made hot-plate pain test instrument to investigate whether *Ganoderma tsugae* increase the pain sensitivity or not. We used 2% formalin by inject into mice paw to observe mice licking response, expecting to see that the mice given *Ganoderma tsugae* will licking for a higher frequency than aging mice. We put 2/3 of mice tail into the hot water (55 °C), then survey whether the tail retract in 15 s, we also want to see that the mice given *Ganoderma tsugae* will retract their tail quicker than aging mice. We let mice stand on the hot-plate instrument for 30 s then record the latency time, await that the latency time of the mice given *Ganoderma tsugae* will shorter than aging mice.

**Conclusions:** Overall, our expected result is that the *Ganoderma tsugae* can increase the pain sensitivity of aging mice.

**Keywords:** Aging, *Ganoderma Tsugae*, Pain, d-Galactose

## A5 The protective effect of artemisinin against oxidative stress in neuronal/non neuronal cells and its underlying mechanisms

### Xin Xigan, Fang Jiankang, Peng Tangming, Wenhua Zheng

#### The Faculty of Heath Sciences, University of Macau, Macau, China

##### **Correspondence:** Wenhua Zheng - WenhuaZheng@umac.mo

*Journal of Chinese Medicine* 2018, **13(Supp 2):**A5

Artemisinin, also known as Qinghaosu (Chinese: 青蒿素) is an anti-malarial drug that possess the most rapid action of all the currently available drugs against Plasmodium falciparum malaria. In past 3 years, we found that artemisinin promoted the survival of various neuronal/non cell types from oxidative insults like H2O2/SNP and beta amyloid. Pretreatment of PC12 cells with artemisinin significantly suppressed SNP/H2O2/Aβ-induced cell death by decreasing the production of intracellular reactive oxygen species (ROS), preventing the decline of mitochondrial membrane potential, restoring abnormal changes in nuclear morphology and reducing LDH release and caspase 3/7 activities in PC12 cells and D407 cells. Artemisinin was able to stimulate the phosphorylation/activation of different signaling protein such as extracellularly regulated protein kinases (ERK) kinase, AMPK and CREB while it had no effect on the Akt pathway. In addition, inhibitor or siRNA of ERK pathway and AMPK attenuated the protective effects of artemisinin whereas the PI3 K inhibitor LY294002 had no effect. Interestingly, intravitreous injection of artemisinin, concentration-dependently reversed the light exposed damage of rat retinal physiological function detected by flash electroretinogram. These results, taken together, suggested that artemisinin is a potential protectant which is able to suppress cell death induced by oxidative stresses. Our results offer support for the potential therapeutic application of artemisinin for prevent and treatment of neuronal degenerative disorders. Supported by NFSC (31771128), FDCT 021/2015/A1, 016/2016/A1 and MYRG2016-00052FSH and MYRG2018-00134-FHS from University of Macau.

## A6 Regulation of oxidative acute lung injury and pulmonary infections by anti-inflammatory reflex

### Sitapara RA^1,2^, Antoine DJ^1,2^, Patel VS^1,2^, Ashby CR Jr.^1,2^, Mantell LL^1,2^

#### ^1^St John’s University College of Pharmacy and Health Sciences, NY, USA; ^2^Feinstein Institute for Medical Research, Northwell Health System, NY, USA

##### Correspondence: Mantell LL - mantell@stjohns.edu, lmantell@northwell.edu

*Journal of Chinese Medicine* 2018, **13(Supp 2):**A6

Oxygen therapy with supraphysiological concentrations of oxygen (hyperoxia) is routinely administered during mechanical ventilation for the management of severe respiratory distress, such as acute respiratory distress syndrome. However, prolonged exposure to hyperoxia results in acute lung injury and compromises the host defense to clear bacteria. Previously, we showed that exposure to hyperoxia can induce the accumulation of alarmin nuclear protein high mobility group box-1 (HMGB1) in the airways. The elevated levels of airway HMGB1 is a critical mediator of both inflammatory lung injury and compromised host defense against bacterial infections in animal models of cystic fibrosis and ventilator-associated pneumonia (VAP). We investigated the effect of GTS-21, a selective α7 nicotinic acetylcholine receptor agonist, on hyperoxia-induced acute lung injury (ALI) and pulmonary infections using mouse models of ALI and VAP. We show here that GTS-21 dose dependently attenuated hyperoxia-induced lung injury characterized by a significant decrease in protein leakage into the airways and pronounced leukocyte infiltration both in the airways as well as lung interstitium. This protective effect of GTS-21 is associated with a significant decrease in hyperoxia-induced accumulation of HMGB1 in the airways. Intriguingly, although inflammation was dampened by the treatment with GTS-21, bacterial clearance in the airways and the lungs was markedly improved. Moreover, hyperoxia-compromised macrophage function in phagocytosis was enhanced by GTS. Our results indicate that GTS-21 is effective in improving bacterial clearance and reducing acute lung injury by enhancing macrophage function via inhibiting the release of nuclear HMGB1 through the reduction of hyperacetylation and oxidation. Therefore, the α7 nicotinic acetylcholine receptor represents a possible pharmacological target to improve the clinical outcome of patients on ventilators by augmenting host defense against bacterial infections.

## A7 Redox imbalance and oxidative stress in diabetes

### Liang-Jun Yan

#### Department of Pharmaceutical Sciences, UNT System College of Pharmacy, University of North Texas Health Science Center, Fort Worth, TX, 76107

##### **Correspondence:** Liang-Jun Yan - liang-jun.yan@unthsc.edu

*Chinese Medicine* 2018, **13(Supp 2):**A7

Cellular redox imbalance refers to the perturbation of the balance between NAD and NADH that are involved in metabolism, cell signaling and stress management. While this NADH/NAD redox imbalance has been implicated in the pathogenesis of diabetes and its implications, the detailed underlying mechanisms are yet to be elucidated. We hypothesize that NADH/NAD redox imbalance with excessive NADH generated by persistent hyperglycemia can overload mitochondrial electron transport chain, in particular complex I (NADH/ubiquinone oxidoreductase), leading to subsequent induction of elevated mitochondrial production of reactive oxygen species (ROS) that then results in increased cell death and accentuation of diabetes and its complication. Using both type 1 and type 2 diabetic animal models, we have proven the correctness of this hypothesis in several organs in that complex I is hyperactive in response to NADH overload and both oxidative damage and cell death are increased. Our study points to the possibility of restoring NADH/NAD redox balance as potential approaches to fighting diabetes and its complications.

## A8 Loganin prevents peripheral nerve injury-induced neuropathic pain via the modulation of TNF-a/NF-kB signaling pathway

### Bin-Nan Wu^1^, Su-Ling Hsieh^2^, Yu-Chin Chang^1^

#### ^1^Department of Pharmacology, Graduate Institute of Medicine, College of Medicine, Kaohsiung Medical University, Kaohsiung, 807, Taiwan; ^2^Department of Pharmacy, Kaohsiung Medical University Hospital, Kaohsiung, 807, Taiwan

##### **Correspondence:** Bin-Nan Wu - binnan@kmu.edu.tw

*Journal of Chinese Medicine* 2018, **13(Supp 2):**A8

**Background:** Neuropathic pain, largely resulting from primary lesions in the peripheral nerve or from malfunctions in the central nervous system, has an extremely negative impact on the quality of life of patients affected by this condition. The chronic constriction injury (CCI) model of peripheral nerve injury has provided a deeper understanding of nociception and the events contributing to the pathogenesis of chronic pain conditions. Loganin is isolated from *Corni fructus*, a well-known herb with glucose-lowering action and neuroprotective activity. This study aimed to investigate the molecular mechanisms of loganin in a rat model of CCI-induced neuropathic pain.

**Methods:** Sprague–Dawley rats were randomly divided into four groups: sham, sham + loganin, CCI and CCI + loganin. Loganin (5 mg/kg/day) was injected intraperitoneally starting at day 1 after surgery. Mechanical and thermal responses were assessed before surgery and at day 3, 7, 14 after CCI. Proximal and distal sciatic nerves (SNs) were isolated for western blots, confocal microscopy and enzyme-linked immunosorbent assay to analyze protein expression, immunoreactivity and proinflammatory cytokines.

**Results:** Behavior data show that thermal hyperalgesia and mechanical allodynia were reduced in loganin treated group as compared to CCI group. The neurobehavioral changes was correlated with the demyelination of Schwann cells, particularly in the distal stump of injured SN. Inflammatory proteins (p-NF-kB, p-IkB, iNOS) and proinflammatory cytokines (TNF-a, IL-1b) induced by CCI were attenuated in the loganin treated group at day 7 after CCI. Loganin also blocked IkB phosphorylation (p-IkB). Double immunofluorescent staining further demonstrated that p-NF-kB protein was reduced by loganin in peripheral glial cells at day 7 after CCI.

**Conclusion:** Based on these findings, we concluded that loganin has antiinflammatory and antihyperalgesia properties in CCI-induced neuropathic pain via decreases in TNF-a/NF-kB activation.

**Acknowledgements:** This study was supported by grant from the Ministry of Science and Technology (MOST 106-2320-B-037-009-MY3), Taiwan.

## A9 Taiwan database of extracts and compounds (TDEC)

### Juan-Cheng Yang^3^, Guan-Yu Chen^3^, Tsung-Yu^4^, Chia-Lin Lee Yao^2^, Yang-Chang Wu^1^

#### ^1^Graduate Institute of Natural Products/Research Center for Natural Products and Drug Development, Kaohsiung Medical University, Kaohsiung, Taiwan; ^2^Department of Cosmeceutics, China Medical University, Taichung, Taiwan; ^3^Chinese Medicine Research and Development Center, Taichung, Taiwan; Kaohsiung Medical University, Kaohsiung, Taiwan

##### **Correspondence:** Chia-Lin Lee Yao - yachwu@kmu.edu.tw; Yang-Chang Wu - chlilee@mail.cmu.edu.tw

*Journal of Chinese Medicine* 2018, **13(Supp 2):**A9

Taiwan database of extracts and compounds (TDEC) is an academic and scientific website which offers a platform for investigators in different fields to share their own research information. Furthermore, the most significant aim of TDEC is to preserve the Taiwan’s important resources, including crude extracts, pure natural isolates, and chemically synthesized derivatives from Chinese Herbal Medicines, marine organisms, and microbes etc. To integrate aforementioned substances from the academic research institutions, industrial units, and botanic conservation centers to make more drugs and products developments efficiently.

TDEC provides functional services such as drug management, drug search, investigator matching, and data statistics systems etc. Drug information consisted of compounds’ structures, physical and chemical properties, and biological activities etc. could be shared for every investigators in the world. Especially for the matching function, it could offer a point-to-point link between drug providers and investigators to efficiently help them to cooperate with each other and promote the powerful researches.

TDEC is a new conceptual data sharing platform for drug researches and developments. So far, this platform is developing under system construction. On the timeline, α version will be online at 2019. (https://tdec.kmu.edu.tw).

**Keywords:** Taiwan database of extracts and compounds, Extracts, Compounds

**Acknowledgements:** TDEC was supported by the grants from Ministry of Science and Technology (MOST 106-2321-B-037-004-)(MOST 107-2321-B-037-004-), Taiwan awarded to Y.C. Wu; We are grateful to the Office of Library and Information Services (OLIS), Kaohsiung Medical University, Taiwan provides technical support.

## A10 Novel Cordyceps vinegar functions as free radical scavenger

### Lu Liu^1^, Humin Fu^2,3^, Yong Hu^1^, Shenghan Lai^4^, Chao Wang^1^, Jun Wang^2,3,4^

#### ^1^Key Laboratory of Fermentation Engineering (Ministry of Education), Hubei Key Laboratory of Industrial Microbiology, Hubei Provincial Cooperative Innovation Center of Industrial Fermentation, Hubei University of Technology, Wuhan, 430068, China; ^2^Department of Pharmacology, School of Food and Biological Engineering, Hubei University of Technology, Wuhan, Hubei, 430068, China; ^3^National 111 Center for Cellular Regulation and Molecular Pharmaceutics, Wuhan, Hubei, 430068, China; ^4^Department of Pathology, Johns Hopkins University School of Medicine, Baltimore, MD, 21287, USA

##### **Correspondence:** Jun Wang - jun_wang@hbut.edu.cn

*Journal of Chinese Medicine* 2018, **13(Supp 2):**A10

**Background:** Free radical serves as a “double-blade” sword for human immune system [1]. Imbalance of free radical causes severe diseases including cardiovascular diseases, cancer, diabetes, and so on [2]. Antioxidants are known to defend cells against damages caused by free radicals [3]. Preventative medicine has obtained more and more attention, including functional food that can provide antioxidants [4]. Rice vinegar is one of the most widely used condiments on earth, especially in the traditional Chinese diet [5]. However, antioxidants have been rarely reported in rice vinegar [6, 7].

**Results:** In this study, we developed one new method to ferment vinegar with treatment of *Cordyceps militaris* (*C. militaris*) solid medium. This cultivated medium is usually discarded, resulting in a large waste of rice resources. In fact, the medium contains the mycelium of *C. militaris* and roots of fruiting bodies in addition to rice, which has extremely high nutritional value. A new process has been applied to brew liquid vinegar by reusing this waste medium. Compared with traditional rice vinegar, the concentration of total flavones (0.810 mg RE/mL), total phenolics (0.451 mg GAE/mL) and adenosine (614.4 μg/mL) in this new Cordyceps vinegar (2.86% acetic acid) was significantly increased. The antioxidant capacity of Cordyceps vinegar was identified by monitoring DPPH free radical clearance and FRAP iron ion reduction. On the other hand, this vinegar was found to contain high concentration of cordycepin (19.64 μg/mL), which hasn’t been reported in other rice vinegars and might contribute to its health benefits as well.

**Conclusion:** Therefore, this Cordyceps vinegar may help remove extra harmful free radicals in human body, which deserves further investigation. In addition, it may benefit human health with its rich nutritional ingredients.

References

1. Bocci V, Valacchi G. Free radicals and antioxidants: how to reestablish redox homeostasis in chronic diseases? Curr Med Chem. 2013;20(27):3397–415.
2. Dreher D, Junod AF. Role of oxygen free radicals in cancer development. Eur J Cancer. 1996;32(1):30–8.
3. Lobo V, Patil A, Phatak A, Chandra N. Free radicals, antioxidants and functional foods: impact on human health. Pharmacogn Rev. 2010;4(8):118–26.
4. Viuda-Martos M, Ruiz-Navajas Y, Fernández-López J, Pérez-Álvarez JA. Spices as functional foods. Crit Rev Food Sci Nutr. 2011;51(1):13–28.
5. Liu D, Zhu Y, Beefink R, Ooijkaas L, Rinzema A, Chen J, Tramper J. Chinese vinegar and its solid-state fermentation process. Food Rev Int. 2004;20(4):407–24.
6. Chou CH, Liu CW, Yang DJ, Wu YH, Chen YC. Amino acid, mineral, and polyphenolic profiles of black vinegar, and its lipid lowering and antioxidant effects in vivo. Food Chem. 2015;168:63–9.
7. Wei K, Cao X, Li X, Wang C, Hou L. Genome shuffling to improve fermentation properties of acetic acid bacterium by the improvement of ethanol tolerance. Int J Food Sci Technol. 2012;47(10):2184–89.

## A11 Aβ_1-42_ oligomers disrupted bEnd.3 brain endothelial cell junction integrity: a potential in vitro model to study TCM protective compounds for blood–brain barrier in Alzheimer’s pathology

### Erin Qian Yue, Xinhua Zhou, Linmin Chen, Maggie Pui Man Hoi

#### State Key Laboratory of Quality Research in Chinese Medicine and Institute of Chinese Medical Sciences, University of Macau, Macau, China

##### **Correspondence:** Maggie Pui Man Hoi - maghoi@umac.mo

*Journal of Chinese Medicine* 2018, **13(Supp 2):**A11

**Background:** Blood–brain barrier (BBB) is an important gatekeeper for homeostasis and exchange of substances in and out of the brain. Brain endothelial cells (BECs) together with astrocytes and pericytes function as the neurovascular unit (NVU) and is critical for BBB physiology. The permeability of BBB is regulated by two types of endothelial cell–cell junctions, tight junctions (TJs) and adherens junctions (AJs). Disruption of BBB integrity is implicated in many neurodegenerative diseases such as Alzheimer’s disease (AD). It is suggested that healthy function of the BBB is important for the clearance of amyloid-beta protein (Aβ). In addition, insults including reactive oxygen species (ROS) and Aβ might induce damages in BBB resulting in deleterious cycle. In recent years, soluble Aβ oligomers rather than the insoluble aggregates are suggested to be the toxic species that cause neuronal cell death. The present study aimed to establish a cellular model of BBB and to evaluate the effects of Aβ on the endothelial cell–cell junctions.

**Method:** We employed immortalized mouse brain endothelial cell (bEnd.3) and mouse astrocyte (C8-D1A) to construct BBB monolayer and co-culture system in vitro. Soluble Aβ_1-42_ oligomer was prepared by dissolving in DMEM supplemented with 0.5% FBS and 1% penicillin–streptomycin and was stored at − 4 °C for 24 h. Cells were incubated with Aβ_1-42_ oligomers (5–40 μM) for 24 h. The integrity of the BBB model was evaluated by Trans Endothelial Electrical Resistance (TEER) and permeability of FITC-conjugated dextran. Proteins of TJs (ZO-1, Claudin-5), AJs (PECAM-1), and cytoskeleton (F-actin) were assessed by immunofluorescence staining.

**Fig. 1:**
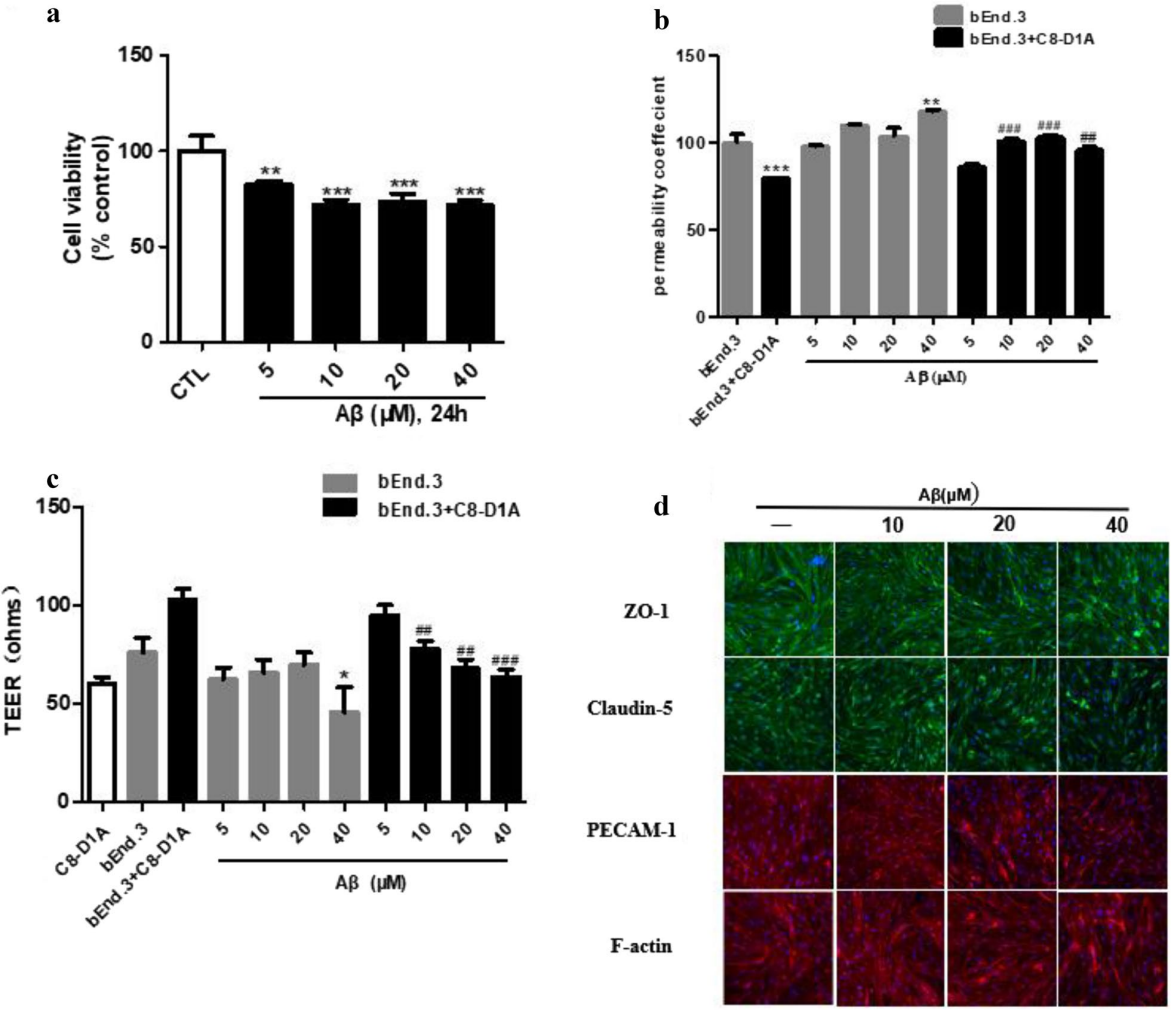
Cell viability of bEnd.3 cell under Aβ_1-42_ oligomers treatment (**a**). Permeability and TEER in bEnd.3 monoculture and co-culture with C8-D1A cells **(b**, **c)**. Immunofluorescent staining of tight junction proteins (ZO-1, Claudin-5), adherens junction protein (PECAM-1), and cytoskeleton F-actin **(d)**. Data represent mean ± SEM of three independent experiments each performed in triplicate (n = 3). *P < 0.05 vs bEnd.3 group; #*P *< 0.05 vs co-culture group. Cells were imaged by IN Cell Analyzer 2000 system (general electric) and analyzed by ImageJ software

**Result and conclusion:** Aβ_1-42_ oligomers exerted significant cytotoxicity on bEnd.3 cells (Fig. 1a) and disrupted BBB functions in both monolayer and co-culture system (Fig. 1b, c). Aβ_1-42_ oligomers also disrupted the expression and arrangement of cell junction proteins and cytoskeleton (Fig. 1d).

**Keywords:** Brain microvascular cell (BMEC), blood–brain barrier (BBB), amyloid-beta_1-42_ (Aβ_1-42_), tight junctions (TJs), adherens junctions (AJs), drug screening.

**Acknowledgements:** This work was supported by Research Committee of University of Macau (MYRG2015-00161-ICMS-QRCM, MYRG2017-00150-ICMS), Macau Science and Technology Development Fund (FDCT/127/2014/A3), and National Natural Science Foundation of China (NSFC81403139).

